# Research on Electrochemical Responses of Lithium-Ion Battery for 3C Consumer Electronics Under Structure Damage

**DOI:** 10.3390/molecules31142505

**Published:** 2026-07-17

**Authors:** Jingyu Yang, Yanru Chen, Rundong Yan, Jiangwei Peng, Jialong Zhao, Xiong Shu, Zixu You, Shangbin Wei

**Affiliations:** 1 Hunan Engineering Technology Research Center for New Fiber Fabric and Processing, Hunan Institute of Engineering, Xiangtan 411104, China; 2 Key Laboratory of Intelligent Textile Processing Technology, Hunan Institute of Engineering, Xiangtan 411104, China; 3 School of Art Design and Media, Sanda University, Shanghai 201209, China; 4 Faculty of Engineering, University of Nottingham, University Park, Nottingham NG7 2RD, UK; 5 Hunan Provincial Key Laboratory of Vehicle Power and Transmission System, Hunan Institute of Engineering, Xiangtan 411104, China

**Keywords:** lithium-ion battery, electrochemical impedance spectroscopy, structure damage

## Abstract

Cylindrical LiFePO_4_ batteries are increasingly used in 3C consumer electronics, including computers, communication devices, and consumer electronic products, owing to their favorable safety characteristics, structural robustness, and stable electrochemical performance. Nevertheless, these batteries may inevitably experience mechanical deformation during manufacturing, transportation, assembly, accidental dropping, collision, or vibration, which can compromise their structural integrity and trigger coupled electrochemical degradation. In this study, the structural-damage-induced failure behavior of cylindrical LiFePO_4_ batteries for 3C consumer electronics was systematically investigated at 25 °C under different states of charge. By integrating mechanical response, in situ open-circuit voltage, surface temperature, and electrochemical impedance spectroscopy, the evolution of electro-mechanical failure during external loading was quantitatively characterized. The results reveal a pronounced State-of-Charge (SOC) dependent failure mechanism: the initial yield load increases with increasing state of charge, indicating improved resistance to mechanical deformation, whereas electrical failure occurs earlier at higher states of charge, accompanied by abrupt voltage collapse, abnormal voltage rebound, and unstable voltage oscillations. This phenomenon demonstrates a clear decoupling between mechanical strength and electrochemical stability under structural damage, suggesting that a higher state of charge enhances the apparent load-bearing capability while simultaneously aggravating internal electrical instability. These findings indicate that mechanical deformation thresholds alone are insufficient for evaluating the safety of LiFePO_4_ batteries used in 3C consumer electronics, and that state of charge, voltage evolution, thermal response, and impedance variation should be jointly considered. This work provides mechanistic insight and experimental guidance for safety assessment, structural protection, and damage-tolerant design of LiFePO_4_ batteries in portable electronic devices and other 3C consumer electronics applications.

## 1. Introduction

Lithium-ion batteries (LIBs) have become a key energy-storage technology for portable electronic devices, electric vehicles, and renewable-energy systems because of their high energy density, long cycle life, and low self-discharge rate [1,2]. Their electrochemical performance is closely related to reversible Li-ion intercalation, electrode chemistry, electrolyte properties, and cathode structure. Layered, spinel, and olivine cathode materials exhibit different characteristics in terms of capacity, operating voltage, thermal stability, rate capability, and cycling durability, while strategies such as elemental doping, surface coating, particle-size control, and structural optimization have been widely employed to improve ionic/electronic conductivity and interfacial stability [3,4,5]. However, with the continuous expansion of LIB applications, their safety and reliability under mechanical abuse have attracted increasing attention. During practical operation, batteries may experience impact, penetration, extrusion, or compression, resulting in casing deformation, electrode damage, impedance variation, voltage fluctuation, localized heating, internal short circuits, and, under severe conditions, thermal instability [6,7,8]. Therefore, understanding the coupled mechanical, electrical, and thermal responses of LIBs under compression is essential for battery safety evaluation and abuse-tolerant design.

Previous studies have investigated the mechanical abuse behavior of LIBs under quasi-static compression, dynamic loading, indentation, puncture, and different battery formats [6,7,8,9,10,11,12,13,14,15,16,17]. For example, compression and indentation tests have been used to analyze thermal runaway triggering, strain localization, deformation-induced tearing, voltage variation, and temperature evolution [18,19,20,21]. These studies have shown that mechanical loading can strongly affect battery integrity and safety, especially when coupled with internal short circuits or severe deformation. However, many of these studies mainly focused on final failure phenomena or thermal runaway behavior, rather than the progressive electrochemical response during structural damage. State of charge (SOC) is another key factor influencing the mechanical and safety behavior of LIBs. Previous studies have shown that higher SOC can increase structural stiffness, accelerate voltage drop, aggravate internal short-circuit behavior, and intensify temperature rise under mechanical loading [22,23,24]. In addition, researchers have developed experimental methods and mechanical models to evaluate force response, deformation characteristics, impedance change, and post-abuse performance under different loading rates, loading directions, and cell chemistries [25,26,27,28,29,30,31,32,33,34,35,36,37]. These studies provide valuable insights into mechanical deformation, electrochemical degradation, internal short circuits, and thermal instability of LIBs.

Nevertheless, several limitations remain. First, many existing studies focus on pouch cells, prismatic cells, or general LIB systems, while the compression-induced failure behavior of cylindrical lithium iron phosphate (LFP) batteries has not been sufficiently clarified. Second, mechanical deformation, electrical response, and thermal behavior are often analyzed separately, and the coupled relationship among structural deformation, voltage variation, impedance evolution, temperature rise, and failure characteristics remains unclear. Third, the progressive electrochemical response of cylindrical LFP cells during stepwise compression has received limited attention.

Therefore, this work focuses on commercially available 18650 cylindrical LFP cells and systematically investigates their mechanical, electrical, thermal, and impedance responses under compression-induced structural damage. The novelty of this study lies in revealing the coupled mechanical–electrical–thermal failure evolution of cylindrical LFP batteries by correlating deformation behavior, voltage response, surface temperature variation, and electrochemical impedance evolution. The results provide reference information for understanding the safety behavior of small-format cylindrical LIBs under structural intrusion or compression conditions and offer guidance for battery safety evaluation, structural design, and abuse-tolerance improvement.

## 2. Method and Test Rig

To investigate the failure characteristics and microstructural evolution of LiFePO_4_ (LFP) cells subjected to structural mechanical damage, eight commercially available 18650 cylindrical LFP batteries (Shenzhen Wotema Battery Co., Ltd., Shenzhen, China) were selected as test specimens, with their key specifications summarized in Table 1. Prior to the formal mechanical abuse experiments, two cells were used for preliminary tests to verify the reliability of the experimental setup and to calibrate the testing equipment, including the compression system, force sensor, displacement measurement system, voltage acquisition system, and temperature monitoring devices. The formal experiments were conducted only after the deviation of the measured parameters was confirmed to be less than 2%, indicating that the equipment and data acquisition system were stable and reliable.

**Figure 1:**
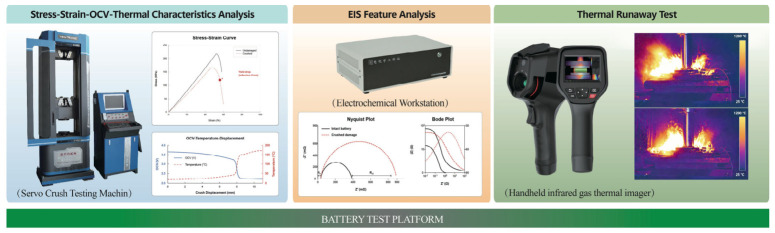
Schematic diagram of the experimental.

The remaining six cells were used for the formal compression tests. Three SOC levels were considered in this study, and two cells were tested at each SOC level to evaluate experimental repeatability. Thus, each SOC condition included two repeated measurements, and the corresponding results were analyzed to assess the consistency of the force–displacement response, voltage variation, temperature evolution, and failure characteristics. The overall experimental configuration is illustrated in Figure 1.

Before the experiments, the force sensor (Shanghai Zhijian Measurement & Control Co., Ltd., Shanghai, China), displacement sensor (Shanghai Zhijian Measurement & Control Co., Ltd., Shanghai, China), voltage acquisition system (Nanjing Qihuo Technology Co., Ltd., Nanjing, China), and thermal imaging camera (Delixi Electric Co., Ltd., Yueqing, China) were calibrated according to the manufacturers’ specifications. The cells were charged or discharged to the target SOC and then allowed to rest under open-circuit conditions until the voltage stabilized. Electrochemical impedance spectroscopy (EIS) was conducted using an electrochemical workstation to characterize the electrochemical response of the cells during progressive mechanical damage. The EIS measurements were performed over a frequency range of 10 kHz to 0.1 Hz with an AC perturbation amplitude of 5 mV at 25 °C. The impedance spectra were analyzed using the equivalent circuit diagram of a lithium iron phosphate battery equivalent circuit model. If no equivalent circuit fitting was applied, the EIS data were used mainly for comparative analysis of impedance evolution during compression.

**Figure 2:**
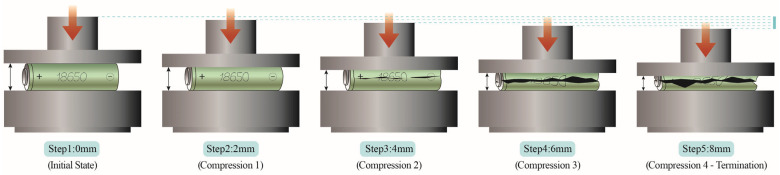
Extrusion testing process.

A thermal imaging camera was used to record the real-time surface temperature distribution of the cells, allowing the thermal response to be correlated with different deformation stages. Meanwhile, a temperature chamber (Guangdong Koming Environmental Instrument Industry Co., Ltd., Dongguan, China) was used to maintain a controlled ambient environment throughout the tests. Mechanical loading was applied using a SASTest universal electronic compression machine (Houxi Pressure Machinery & Equipment Co., Ltd., Zhengzhou, China) under quasi-static compression conditions. The crosshead speed was set to 1 mm/min. During the extrusion tests, the cell was incrementally compressed in 2 mm displacement steps, and the loading was paused at each step for EIS acquisition. This procedure allowed the evolution of impedance spectra to be tracked as mechanical damage accumulated. The test was terminated when the total deformation reached 8 mm. The detailed stepwise procedure is shown in Figure 2.

## 3. Results and Analysis

**Figure 3:**
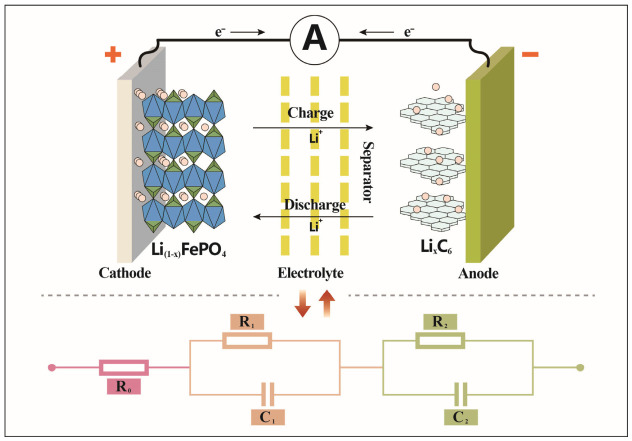
Equivalent circuit diagram of lithium iron phosphate battery.

Electrochemical impedance spectroscopy (EIS) was conducted to evaluate the electrochemical response of cylindrical LiFePO_4_ batteries under mechanical extrusion. EIS can reveal the changes in ohmic resistance, interfacial polarization, charge-transfer behavior and lithium-ion diffusion by measuring the impedance response of the battery over a wide frequency range. In this study, the impedance spectra were further analyzed by Equivalent circuit diagram of lithium iron phosphate battery to obtain quantitative parameters rather than relying only on visual comparison of Nyquist plots. As shown in Figure 3, the equivalent circuit consists of ohmic resistance R_0_, interfacial film resistance R_1_, charge-transfer resistance R_2_, constant phase elements C_1_ and C_2_, and a diffusion-related Warburg element. R_0_ represents the ohmic resistance of the electrolyte, current collectors, tabs and external connections, which is mainly reflected by the high-frequency intercept. R_1_ is associated with the resistance of the solid electrolyte interphase (SEI) and other interfacial films at the electrode/electrolyte interface. C_1_ is introduced to describe the non-ideal capacitive behavior of the heterogeneous interfacial layer. R_2_ corresponds to the charge-transfer resistance, while C_2_ reflects the non-ideal double-layer capacitance related to the charge-transfer process. The Warburg element represents the diffusion-controlled transport of lithium ions in the electrode materials, especially in the low-frequency region. After extrusion, the fitted curves show clear variations.

### 3.1. EIS Features

**Figure 4:**
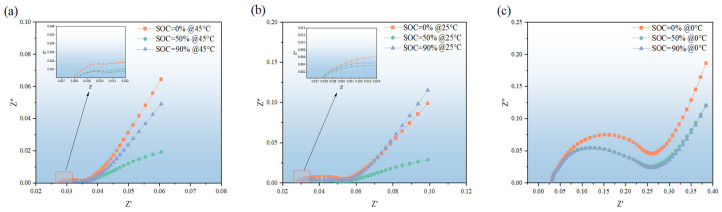
EIS characteristics of LFP batteries under different SOCs and temperatures before extrusion. (**a**) EIS curves for different SOCs at 45 °C; (**b**) EIS curves for different SOCs at 25 °C; (**c**) EIS curves for different SOCs at 0 °C.

Electrochemical impedance spectroscopy (EIS) is a nondestructive, highly sensitive, and high-resolution frequency-domain diagnostic technique that can capture the kinetic signatures of multiple internal electrochemical processes in LIBs. However, the measured impedance response is not uniquely determined by degradation or failure alone, but is also strongly modulated by operating conditions such as temperature and state of charge (SOC), which may obscure or even mimic the impedance variations induced by mechanical damage. Therefore, before interpreting extrusion-triggered failure features using EIS-derived indicators, baseline measurements were first conducted on intact cells (without compression) over a matrix of SOCs and ambient temperatures. Figure 4 shows the test results of the effects of different temperatures and SOCs on the EIS of LFP batteries before extrusion. The experiments were conducted at ambient temperatures of 0 °C, 35 °C, and 45 °C, with SOCs are 0%, 50%, and 90%. Figure 4a–c display the EIS curves under different SOCs at 45 °C, 25 °C, and 0 °C, respectively.

Figure 4 provides a baseline map of the whole-frequency (i.e., the frequency range is 0.01 Hz–10 KHz) EIS response of intact 18650 LFP cells measured under a matrix of three ambient temperatures (0 °C, 35 °C, 45 °C) and three SOC levels (0%, 50%, 90%).

In the high-frequency region, the impedance response is mainly reflected by the ohmic resistance, which can be evaluated from the high-frequency intercept on the real axis and further quantified by the fitted Rs value. As shown in Figure 4a–c, the high-frequency intercept generally shifts to higher resistance at 0 °C, suggesting increased ohmic resistance due to reduced ionic conductivity and slower electrolyte transport at low temperature. At elevated temperature, the intercept remains relatively low, indicating improved bulk ionic transport. However, no definitive conclusion regarding thermally induced interfacial instability can be drawn from the high-frequency intercept alone. Therefore, the temperature-related changes are discussed here primarily in terms of the measured Rs variation rather than as direct evidence of specific interfacial degradation mechanisms. Within each temperature condition, the high-frequency regions of the spectra at different SOCs show relatively small differences compared with the temperature-induced variation. This suggests that temperature has a stronger influence on the ohmic resistance than SOC under the present testing conditions. In contrast, SOC-dependent effects are more evident in the medium- and low-frequency regions, where interfacial polarization and diffusion-related processes contribute more significantly to the impedance response. This temperature–SOC baseline is useful for distinguishing subsequent impedance changes caused by mechanical compression from those caused by operating-condition variations.

In the mid-frequency region, the Nyquist plots exhibit a semicircular feature. In the revised interpretation, this semicircle is not attributed solely to charge-transfer resistance. Instead, it reflects the combined contribution of SEI-related resistance, interfacial contact resistance, charge-transfer resistance, and non-ideal capacitive behavior. The fitted R_SEI_ and Rct parameters provide further support for this interpretation. As temperature decreases, both R_SEI_ and Rct tend to increase, indicating that low temperature may weaken interfacial ion transport and slow down electrochemical reaction kinetics. Among these parameters, Rct usually shows a more pronounced temperature dependence, suggesting that the charge-transfer process is particularly sensitive to thermal conditions. At elevated temperature, the reduction in Rct and R_SEI_ indicates improved interfacial reaction activity and lower polarization resistance. The SOC-dependent impedance variation is mainly reflected in the interfacial parameters rather than in Rs. The fitted Rct generally shows a SOC-dependent trend, with relatively higher values at low or high SOC and lower values at intermediate SOC. This suggests that the electrochemical reaction activity may be more favorable at moderate SOC, whereas extremely low or high SOC can lead to stronger interfacial polarization. Meanwhile, variations in R_SEI_ and C-related parameters indicate that SOC may also influence the interfacial film response and non-ideal capacitive behavior. However, these changes should be interpreted as impedance-response-based indications rather than direct evidence of specific SEI structural evolution.

In the low-frequency region, the EIS response is predominantly governed by lithium-ion transport, and the impedance tail therefore reflects diffusion-related limitations (i.e., the Warburg-type behavior) associated with concentration gradients and effective diffusivity. As illustrated in Figure 4a–c, the slope and extent of the low-frequency tail vary systematically with both SOC and ambient temperature, indicating that diffusion polarization is highly sensitive to operating conditions. According to Figure 4a–c, the tail slope reaches its minimum at 50% SOC, whereas the slopes at 0% and 90% SOC are comparable to each other and are markedly larger than that at 50% SOC. This trend indicates that diffusion-related impedance is lowest at intermediate SOC and becomes more severe at both SOC extremes. At 50% SOC, the electrode typically operates in a comparatively balanced state, where the availability of intercalation sites and the local Li^+^ concentration are moderate, leading to weaker concentration polarization and more uniform Li^+^ distribution; consequently, the diffusion impedance is minimized, and the low-frequency tail becomes less steep. In contrast, at 90% SOC, the electrode approaches a highly lithiated state, where site saturation and reduced chemical diffusion can intensify concentration gradients near the interface, giving rise to stronger diffusion polarization and a steeper low-frequency tail. Notably, a similarly steep tail is also obtained at 0% SOC. Under deep delithiation, the scarcity of Li^+^ within the host structure and the reduced ionic inventory near the interface can likewise generate pronounced concentration gradients during perturbation. At the same time, limited percolation of Li^+^ within the solid phase and possible inhomogeneous reaction distribution exacerbate transport resistance. As a result, both SOC extremes (0% and 90%) exhibit comparable and elevated diffusion impedance relative to 50% SOC. Furthermore, comparisons across temperatures (Figure 4a at 45 °C, Figure 4b at 25 °C, and Figure 4c at 0 °C) suggest that lowering the temperature strengthens the diffusion-controlled behavior at all SOCs, consistent with reduced ionic mobility and slower solid-state transport. Importantly, the minimum tail slope at 50% SOC is preserved across the tested temperatures, reinforcing that intermediate SOC provides the most favorable condition for mitigating concentration polarization, whereas diffusion limitations intensify symmetrically toward both low and high SOC boundaries.

Overall, the fitted impedance parameters show that temperature mainly affects Rs, and Rct by regulating electrolyte conductivity and interfacial reaction kinetics, while SOC has a stronger influence on interfacial polarization and diffusion-related impedance. These results provide a more quantitative basis for interpreting the EIS spectra and avoid attributing the observed semicircle variation solely to charge-transfer resistance.

**Figure 5:**
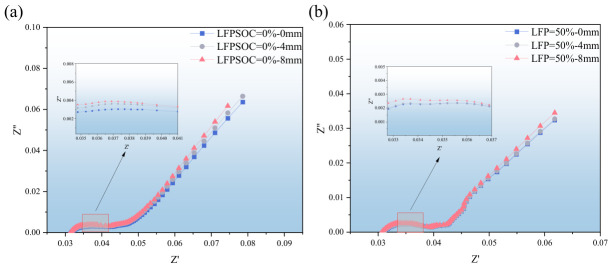

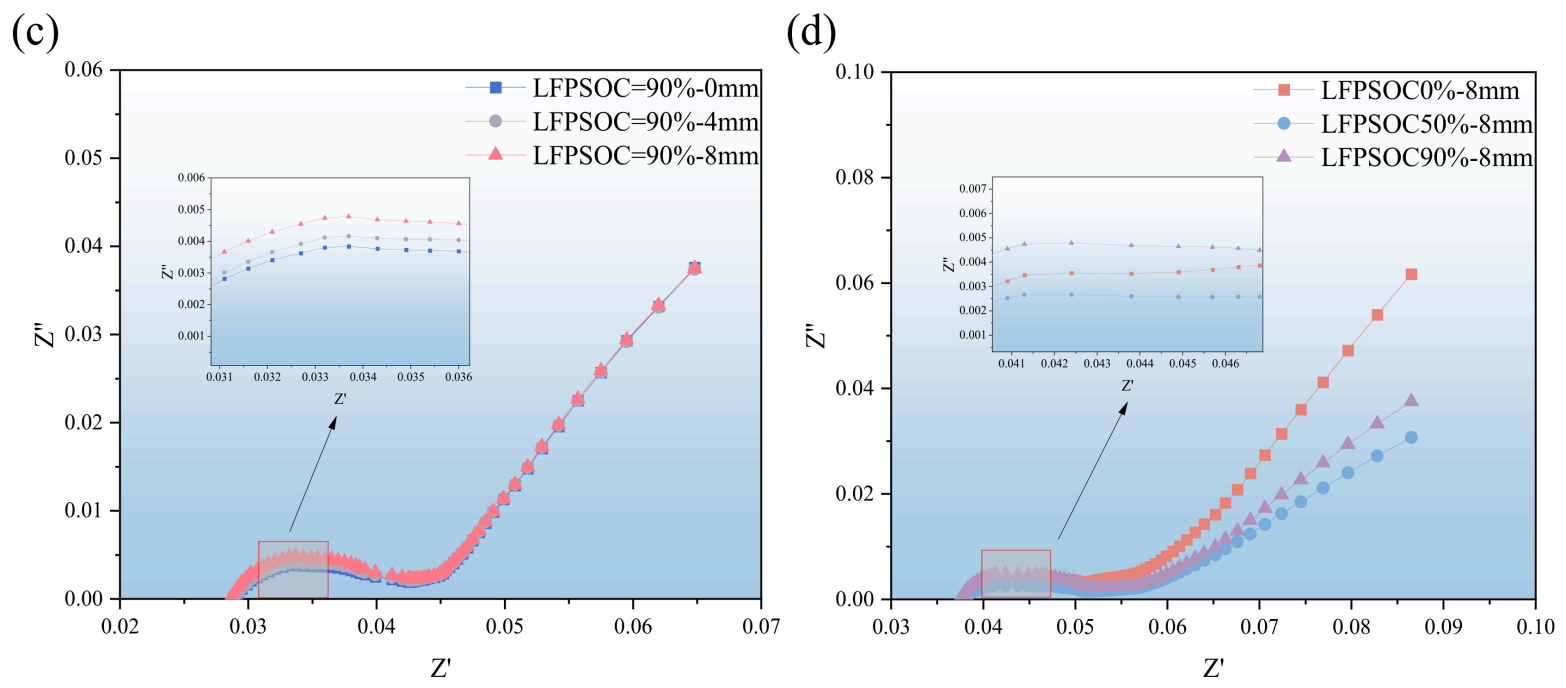
EIS features of LFP batteries under different SOCs and extrusion deformations. (**a**) EIS curves for different deformation with the SOCs is 0%; (**b**) EIS curves for different deformation with the SOCs is 50%; (**c**) EIS curves for different deformation with the SOCs is 90%; (**d**) EIS curves for different SOC with the extrusion deformation is 8 mm.

Given that the above research demonstrates a pronounced dependence of the EIS response on both ambient temperature and SOC, strict control of thermal conditions is required to decouple mechanically induced effects from temperature-driven impedance variations. Therefore, in the following experiments, the EIS characteristics of LIBs subjected to external compression will be systematically investigated under a fixed ambient temperature of 25 °C, and the results are shown in Figure 5.

As shown in Figure 5, the extrusion-induced evolution of the EIS spectra at 25 °C exhibits clear SOC-dependent characteristics and varies across different frequency regions. For the cells at 0% and 50% SOC, as shown in Figure 5a and Figure 5b, respectively, increasing extrusion displacement leads to an overall upward shift in the impedance spectra. Specifically, the high-frequency intercept increases, the mid-frequency semicircle becomes larger, and the low-frequency tail shows an evident upward trend. These changes indicate that mechanical extrusion affects multiple electrochemical processes, including ohmic resistance, interfacial impedance, and diffusion-related impedance. With increasing deformation, the impedance increase becomes more pronounced, suggesting that mechanical compression progressively deteriorates the internal electrochemical response of the cell.

In the high-frequency region, the increased intercept suggests a rise in ohmic resistance, which may be related to deformation-induced changes in internal conductive pathways or contact conditions. In the mid-frequency region, the enlarged semicircle reflects an increase in the overall interfacial impedance, which may involve the combined effects of SEI-related resistance, interfacial contact resistance, charge-transfer resistance and non-ideal capacitive behavior. In the low-frequency region, the elevated diffusion tail suggests that ion transport may become more restricted under compression. However, because no direct post-mortem structural characterization was performed in this study, these changes should be regarded as impedance-response-based indications rather than direct evidence of specific structural damage mechanisms such as electrode densification, separator deformation or particle cracking. In contrast, the cell at 90% SOC exhibits a different impedance response, as shown in Figure 5c. With increasing extrusion displacement, the high- and mid-frequency regions still show obvious changes, whereas the low-frequency response remains relatively stable. This result suggests that, under high-SOC conditions, mechanical deformation mainly influences the ohmic and interfacial impedance components within the tested deformation range, while the diffusion-related response is less sensitive to extrusion. One possible explanation is that the low-frequency impedance at high SOC may already be governed by the existing lithium-ion concentration distribution and electrode state, so additional compression does not produce a clearly measurable change in the diffusion tail under small-signal EIS testing. Meanwhile, deformation may still affect local current pathways and interfacial polarization, resulting in the observed increase in the high- and mid-frequency impedance.

Overall, Figure 5 indicates that the electrochemical response to extrusion is strongly dependent on SOC. At 0% and 50% SOC, compression affects the impedance response across the high-, mid- and low-frequency regions, whereas at 90% SOC the deformation-induced variation is mainly concentrated in the high- and mid-frequency regions. Nevertheless, the underlying structural origins of these impedance changes cannot be fully distinguished based on EIS alone. Further post-mortem analyses, such as SEM, X-ray computed tomography or cross-sectional characterization, are required to clarify whether the observed impedance evolution is mainly caused by contact loss, separator deformation, SEI modification, electrode cracking or other structural effects.

### 3.2. Mechanical-Electrical-Thermal Features

**Figure 6:**
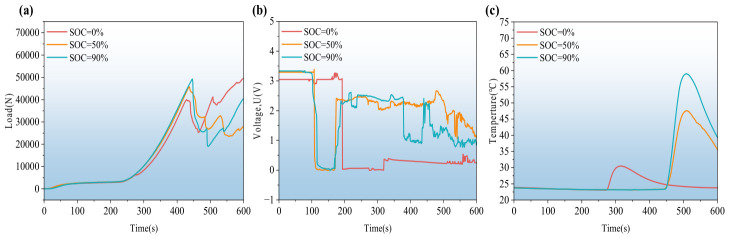
Comparison of stress–strain, voltages and temperatures at different SOCs. (**a**) Stress–strain behavior at different SOCs; (**b**) OCV behavior at different SOCs; (**c**) temperatures behavior at different SOCs.

To further elucidate the failure features of LIBs under compressive loading, subsequent experiments will be conducted by extruding the cells at a constant displacement rate of 2 mm min^−1^ while simultaneously monitoring the stress–strain behavior, open-circuit voltage (OCV) evolution, and surface temperature response. Considering that lithium-ion batteries are predominantly operated under room-temperature conditions in practical applications and that their electrochemical state can vary substantially, all tests will be performed at a controlled ambient temperature of 25 °C with the state of charge set to 0%, 50%, and 90%, respectively. The corresponding results are shown in Figure 6.

Figure 6 compares the coupled mechanical, electrical, and thermal responses of the cells during extrusion at different SOCs. 

As shown in Figure 6a, the first yield point appears earliest for the cell at 0% SOC, with the lowest corresponding load of approximately 38 kN. The 50% SOC cell yields at a higher load of approximately 48 kN, whereas the 90% SOC cell sustains the highest load before the first yield point, reaching approximately 51 kN. This increasing load-bearing capacity with SOC indicates that the mechanical response of the cell is strongly dependent on its electrochemical state. At higher SOC, lithium is increasingly inserted into the graphite anode, which can cause lattice expansion and lithiation-induced stress within the electrode particles. Meanwhile, the corresponding delithiation of the LFP cathode and the change in electrode volume may alter the internal stress state, particle–binder contact, and electrode/current collector interaction. These SOC-dependent changes reduce the internal void compliance and increase the compactness of the jelly-roll structure, resulting in a higher apparent stiffness and a larger load required to initiate macroscopic yielding.

However, the increased stiffness at high SOC does not necessarily indicate improved mechanical safety. Under compression, the more compact electrode stack may intensify separator compression and reduce the tolerance for local deformation. Once local contact loss, particle fracture, binder failure, or current collector deformation occurs, the local electronic/ionic transport pathways may become non-uniform, leading to current concentration and impedance increase. In addition, severe deformation at high SOC may promote internal short-circuit risk and accelerate local heat generation, which can further induce electrolyte decomposition and reduce electrochemical stability. Therefore, the higher yield load observed at elevated SOC is mainly attributed to increased stack stiffness and internal stress, while the accompanying decrease in electrochemical stability may result from compression-induced separator damage, localized current concentration, and accelerated interfacial degradation.

The OCV responses in Figure 6b exhibit an opposite SOC dependence in terms of electrical failure timing. At 0% SOC, the voltage remains stable for a longer period and then collapses abruptly to 0 V at ~200 s, indicating that an electrically critical event (e.g., a major loss of electrical continuity or an internal short-circuit pathway reaching a percolation threshold) occurs later in the loading sequence. In contrast, for both 50% and 90% SOC, the OCV drops to 0 V much earlier, at approximately ~100 s, with only a small difference between the two. The earlier voltage collapse at higher SOC suggests that charged cells are more prone to mechanically induced electrical breakdown, because the higher stored electrochemical energy and increased electrode reactivity reduce the tolerance to separator damage and local contact failure. Moreover, at elevated SOC, the electrode stack typically exhibits higher interfacial stress sensitivity; therefore, relatively small structural disruptions (e.g., separator thinning/tearing, particle cracking, or current-collector wrinkling) can more readily trigger shorting or abrupt voltage loss.

Thermal responses under extrusion are compared in Figure 6c. At 0% SOC, the surface temperature changes only slightly, suggesting minimal heat generation during compression. At 50% SOC, a clear temperature rise is observed, with an accelerated increase as deformation proceeds, reflecting enhanced irreversible dissipation and potentially additional heat release associated with mechanically triggered electrochemical reactions. At 90% SOC, the temperature rises most rapidly and reaches the highest peak value, substantially exceeding those at 0% and 50% SOC. This pronounced heating is consistent with the larger stored electrochemical energy at high SOC and the greater susceptibility to deformation-induced internal short circuits and exothermic parasitic reactions, thereby significantly elevating the risk of thermal runaway.

Overall, Figure 6 demonstrates that the extrusion-induced failure response of the LIBs intensifies with SOC: low-SOC cells show superior stability, negligible voltage perturbation, and limited heating, whereas high-SOC cells exhibit faster stress build-up, stronger voltage instability, and a markedly amplified thermal response. These results identify the high-SOC condition as the most critical state for mechanical-abuse safety and underscore the need for targeted mitigation strategies under highly charged conditions.

### 3.3. Microstructural Analysis

**Figure 7:**
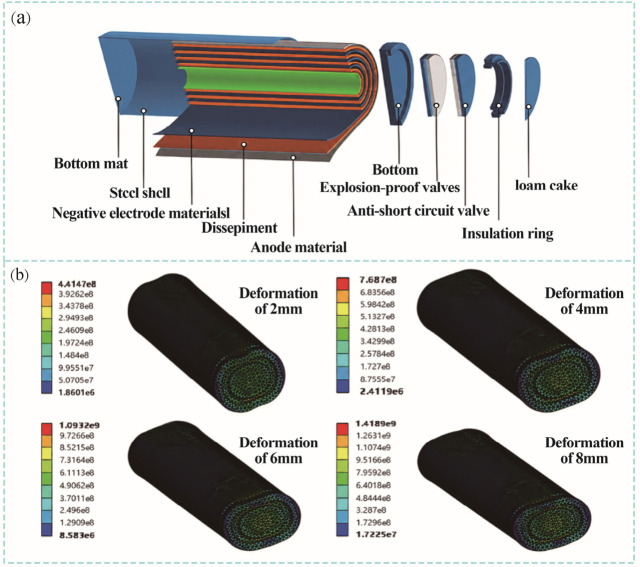
Structure of the 18650 LIBs and stresses under different deformations. (**a**) Structure of the lithium-ion batteries; (**b**) stress diagram under different deformations.

Similar to other cylindrical lithium batteries, the 18650 cylindrical lithium iron phosphate (LFP) battery primarily consists of positive and negative electrode sheets, a separator, electrolyte, tabs, a winding structure, a top cover, and a sealing ring, as shown in Figure 7a. In addition, to analyze the stresses in various parts of the LIBs structure during the extrusion process—to better carry out the subsequent extrusion test—we analyzed the stresses in the LIBs under different extruded deformation conditions from 0 to 8 mm, and obtained the results as shown in Figure 7b.

In Figure 7b, the stresses on the battery structure are relatively concentrated under different extrusion deformation displacements of the lithium-ion battery in the extrusion process, all of which appear in the battery extrusion contact surface, and at the same time, with the increase in the extrusion displacement, the stresses on the lithium-ion battery gradually increase. In order to further verify the failure characteristics of lithium-ion batteries after extrusion deformation, we carried out extrusion tests on multifunctional stress-extrusion equipment for the studied batteries.

**Figure 8:**
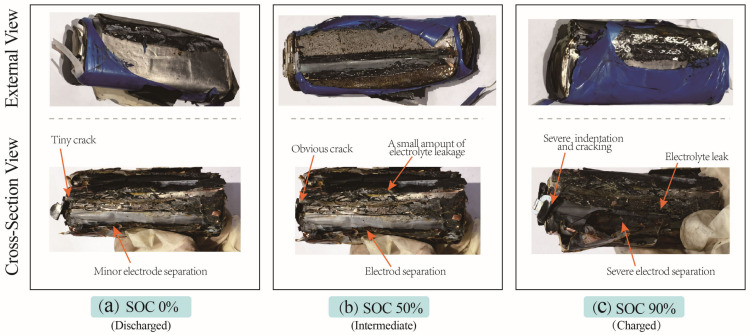
Structural changes in the lithium-ion battery after compression. (**a**) Structural damage after compression when the SOC is 0%; (**b**) structural damage after compression when the SOC is 50%; (**c**) structural damage after compression when the SOC is 90%.

After the compression test, significant structural damage was observed in the battery’s tabs, winding structure, top cover, and sealing ring, as shown in Figure 8.

In Figure 8, the extent of structural damage in LFP batteries varies with different SOC. When the SOC is low, structural damage is relatively minor. Although small cracks appear at the welding points between the tabs and electrode sheets, the overall structure remains intact. The top cover and sealing ring show no noticeable deformation or damage, and no electrolyte leakage is observed. As SOC increases to 50%, structural damage becomes more severe. Notably, the battery’s tabs and winding structure develop more pronounced cracks, and partial detachment occurs at the welding points between the tabs and electrode sheets. The top cover exhibits slight indentations, while the sealing ring undergoes some compression deformation. Minor electrolyte leakage is observed, though not at a critical level. At SOC is 90%, the battery exhibits the most significant structural damage. The external blue plastic film and steel casing rupture, leading to severe electrolyte leakage. Visible cracks and detachment are observed at the welding points of the tabs and electrode sheets. The top cover shows more severe indentation and cracking, while the sealing ring exhibits distinct burn marks, indicating the possible occurrence of thermal runaway at this level of SOC.

Overall, the failure characteristics of LFP batteries under compression vary significantly across different SOC levels. The higher the SOC, the more severe the structural damage, particularly at SOC is 90%, where the battery exhibits structural deformation, welding failures, tab-electrode separation, and signs of thermal runaway. These findings highlight the need for enhanced thermal management and structural design in energy storage batteries at high SOC levels to prevent catastrophic failures under mechanical abuse conditions.

## 4. Conclusions

This study systematically investigated the effects of mechanical compression on the structural integrity, electrochemical stability, and thermal safety of cylindrical LiFePO_4_ batteries at different states of charge from a practical engineering perspective. By correlating mechanical response, open-circuit voltage evolution, surface temperature variation, and electrochemical impedance characteristics, the compression-induced failure behavior and SOC-dependent degradation mechanisms were clarified. The main conclusions are as follows.

(1) Mechanical compression changes the impedance characteristics of LiFePO_4_ batteries. With increasing compression displacement, the fitted EIS parameters show an increase in charge-transfer resistance and diffusion-related impedance, suggesting enhanced interfacial polarization and more restricted lithium-ion transport. The ohmic resistance changes only slightly, indicating that the bulk conductive pathway is less sensitive to moderate compression than the interfacial and diffusion-related processes.

(2) The battery response under compression is affected by SOC. Cells at higher SOC show more pronounced voltage fluctuation, impedance variation and temperature rise than those at lower SOC. These results suggest that high SOC may reduce the tolerance of the cell to mechanical deformation and increase the possibility of internal electrochemical instability. However, detailed structural damage modes, such as separator deformation, particle cracking and contact degradation, still require further post-mortem characterization.

(3) The coupled mechanical, voltage and thermal responses indicate that SOC is an important factor influencing the safety behavior of LiFePO_4_ batteries under compression. Low-SOC cells remain relatively stable during deformation, whereas high-SOC cells exhibit stronger electrochemical disturbance and localized temperature increase. Since thermal runaway was not directly triggered in this study, the observed temperature rise and impedance deterioration are interpreted as indicators of increased safety risk rather than direct evidence of thermal runaway. Therefore, compression safety evaluation should consider not only mechanical deformation but also voltage response, impedance evolution and thermal behavior.

Although this study investigated the electrochemical and thermal responses of cylindrical LiFePO_4_ batteries under mechanical extrusion through voltage monitoring, temperature measurement and EIS analysis, the detailed internal failure mechanisms still need further structural verification. In future work, advanced post-mortem characterization methods, such as scanning electron microscopy (SEM), electrode cross-sectional analysis and X-ray computed tomography, will be employed to further examine separator deformation, electrode particle cracking, interfacial contact degradation and possible internal short-circuit pathways.

## Figures and Tables

**Table 1:** Parameters of LFPs.

Item	Parameters
Capacity ±0.1 Ah	1.25
AC resistance (mΩ)	≤1.6
Weight (g) ±0.5 g	40.1
Operating voltage range	4.2–2.0
Charging allowed temperature Range (°C)	0–45
Discharge allowed temperature range (°C)	−20–50
Continuous operation current	1.5 C
Nominal Voltage (V) ±0.1 V	3.1

## Data Availability

The original contributions presented in this study are included in the article. Further inquiries can be directed to the corresponding author.
